# ERK hyperactivation serves as a unified mechanism of escape in intrinsic and acquired CDK4/6 inhibitor resistance in acral lentiginous melanoma

**DOI:** 10.1038/s41388-023-02900-6

**Published:** 2023-12-08

**Authors:** Kasturee Jagirdar, Marie E. Portuallo, Meihan Wei, Matthew Wilhide, Jeremy A. Bravo Narula, Bailey M. Robertson, Gretchen M. Alicea, Crystal Aguh, Min Xiao, Tetiana Godok, Dylan Fingerman, Gregory Schuyler Brown, Meenhard Herlyn, Vissy M. Elad, Xinyu Guo, Eneda Toska, Daniel J. Zabransky, Bradley Wubbenhorst, Katherine L. Nathanson, Shawn Kwatra, Yogesh Goyal, Hongkai Ji, Qin Liu, Vito W. Rebecca

**Affiliations:** 1 Department of Biochemistry and Molecular Biology, Johns Hopkins Bloomberg School of Public Health, Baltimore, MD USA; 2 Department of Chemical and Biomolecular Engineering, The Johns Hopkins Whiting School of Engineering, Baltimore, MD USA; 3 Department of Dermatology, Johns Hopkins School of Medicine, Baltimore, MD USA; 4 The Wistar Institute, Molecular and Cellular Oncogenesis Program and Melanoma Research Center, Philadelphia, PA USA; 5 Sidney Kimmel Comprehensive Cancer Center and Department of Oncology, Johns Hopkins School of Medicine, Baltimore, MD USA; 6 Department of Medicine, Division of Translational Medicine and Human Genetics, University of Pennsylvania, Perelman School of Medicine, Philadelphia, PA USA; 7 Department of Cell and Developmental Biology, Northwestern University, Feinberg School of Medicine, Chicago, IL USA; 8 Department of Biostatistics, Johns Hopkins Bloomberg School of Public Health, Baltimore, MD USA

**Keywords:** Melanoma, Cancer therapeutic resistance

## Abstract

Patients with metastatic acral lentiginous melanoma (ALM) suffer worse outcomes relative to patients with other forms of cutaneous melanoma (CM), and do not benefit as well to approved melanoma therapies. Identification of cyclin-dependent kinase 4 and 6 (CDK4/6) pathway gene alterations in >60% of ALMs has led to clinical trials of the CDK4/6 inhibitor (CDK4i/6i) palbociclib for ALM; however, median progression free survival with CDK4i/6i treatment was only 2.2 months, suggesting existence of resistance mechanisms. Therapy resistance in ALM remains poorly understood; here we report hyperactivation of MAPK signaling and elevated cyclin D1 expression serve as a mechanism of intrinsic early/adaptive CDK4i/6i resistance. ALM cells that have acquired CDK4i/6i resistance following chronic treatment exposure also exhibit hyperactivation of the MAPK pathway. MEK and/or ERK inhibition increases CDK4i/6i efficacy against therapy naïve and CDK4i/6i-resistant AM cells in xenograft and patient-derived xenograft (PDX) models and promotes a defective DNA repair, cell cycle arrested and apoptotic program. Notably, gene alterations poorly correlate with protein expression of cell cycle proteins in ALM or efficacy of CDK4i/6i, urging additional strategies when stratifying patients for CDK4i/6i trial inclusion. Concurrent targeting of the MAPK pathway and CDK4/6 represents a new approach for patients with metastatic ALM to improve outcomes.

## Introduction

Acral lentiginous melanoma (ALM) constitutes a distinct disease relative to other forms of cutaneous melanoma (CM) (i.e., superficial spreading, nodular, lentigo maligna) due, in part, to a different cell of origin (volar versus non-volar skin melanocytes) [1], the defining acral skin sites they arise on, and a complex genomic landscape [2]. Although patients with metastatic ALM suffer worse outcomes relative to patients with other subtypes of CM, the underlying molecular mechanisms responsible for ALM initiation, progression, and therapy resistance remain poorly understood [3, 4]. Existing standard-of-care targeted therapies (i.e., BRAF inhibitors) for CM are not available to the majority of advanced ALM patients due to a lower frequency of *BRAF*^V600E/K^ mutations (20% versus 50% in other forms of CM [2]). Further, targeted therapy is not as effective in ALM patients with *BRAF*^V600E/K^ mutations [5]. Additionally, the efficacy of immune checkpoint blockade (ICB) is less effective and remains poorly understood in ALM [2, 5, 6]. Therefore, new therapy strategies tailored to the ALM patient population are critically warranted.

Recent genetic characterization of ALM patient tumor tissue has identified cyclin-dependent kinase 4 (CDK4)-pathway (e.g., *CDK4* amplification, *CDK6* amplification*, CCND1* amplification, *P16*^INK4A^ loss) alterations in 53–82% of ALM cases [2, 7], with *CDK4* amplification and *P16*^INK4A^ loss each independently serving as predictors of shorter patient overall survival. CDKs propel cell cycle progression and their frequent dysregulation in cancer contributes to the uncontrolled cellular proliferation, which is regarded as one of the hallmarks of cancer. ALM cell lines and patient-derived xenograft (PDX) models with CDK4 pathway alterations were reported to exhibit elevated in vivo sensitivity to CDK4/6 inhibitors (CDK4i/6i) [7], which provided rationale for the first phase II clinical trial (NCT03454919) of palbociclib in patients with advanced ALM whose tumors exhibit CDK4-pathway aberrations [8]. Unfortunately, most patients did not benefit from palbociclib monotherapy, and the median progression free survival (mPFS) was only 2.2 months. The few patients who did respond did not often experience durable tumor control, suggesting that both intrinsic (early/adaptive)- and acquired-resistance mechanisms to single-agent palbociclib arise. To date, mechanisms leveraged by ALM cells to escape CDK4i/6i remain poorly understood.

Here, we report that hyperactivation of the mitogen-activated protein kinase (MAPK) pathway and elevated cyclin D1 promote intrinsic CDK4i/6i resistance. Mechanistically, CDK4i/6i reduces DUSP4 protein expression that underlies the hyperactivation of ERK. Further, ALM cells with acquired resistance to CDK4i/6i following chronic drug exposure (i.e., >2 months) exhibit elevated ERK activity. The MAPK pathway sustains cyclin D1 levels, and we find in the context of therapy naïve ALM cells, elevated MAPK activity promotes ALM addiction to cyclin D1, which can be overcome with use of the clinical MEK inhibitor trametinib, an ERK inhibitor, or genetic silencing of cyclin D1. Treatment with a MEK inhibitor, ERK inhibitor, or silencing of cyclin D1 also resensitizes ALM with acquired resistance to CDK4i/6i. Altogether, these findings represent the seminal report of an intrinsic and acquired CDK4i/6i resistance mechanism in ALM and conclude the addition of a MEK inhibitor may increase the durability of first- and second-line CDK4i/6i therapy in patients with advanced ALM.

## Results

### Genetic status of CDK4-pathway nodes does not predict protein expression or CDK4i/6i durability in ALM

The current strategy for clinical use of CDK4i/6i in patients with advanced ALM rests upon the genetic status of CDK pathway nodes, in part due to recent evidence that *Cdk4* and/or *P16*^*INK4a*^ copy number status may be of prognostic significance for ALM patients [7]; stemming from this study, only patients with *CDK4* gain, *CCND1* gain and/or *CDKN2A* loss were eligible for treatment with palbociclib [8]. In an independent analysis of a separate ALM patient cohort (*n* = 75 primary samples with survival information) [9], we find no significant correlation between the overall survival of ALM patients with wildtype *CDK4* (*n* = 57) versus *CDK4* gain (*n* = 16), or wildtype *CDKN2A* (*n* = 47) versus *CDKN2A* loss (*n* = 27) (Supplementary Fig. 1A, B).

**Fig. 1:**
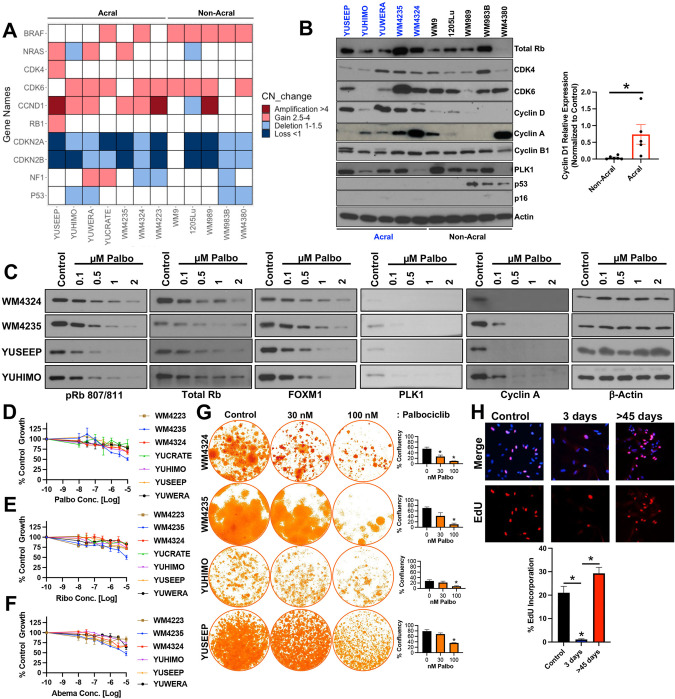
Genetic status of CDK4-pathway nodes does not predict protein expression or durability of CDK4/6 inhibition. **A** Copy number variation and mutational status were assessed across a panel of ALM and non-ALM models. **B** Western blot showing basal expression of cell cycle proteins across a panel of ALM and non-ALM models. Shown in the right panel is a densitometric quantification of cyclin D1 expression. **C** Cells were treated with increasing concentrations of palbociclib for 24 h before Western blotting. **D** A panel of ALM models were treated with increasing concentrations of palbociclib, **E** ribociclib, or (**F**) abemaciclib for 72 h before cell numbers were quantified using MTT. Bars show S.E. mean. **G** A panel of ALM cell lines were treated with palbociclib for 3–4 weeks before colonies were fixed and stained with crystal violet. Photographs are representative of three independent experiments and relative clonogenic survival quantitation is shown to the right. **H** WM4324 cells were treated with palbociclib (500 nM) for the time shown before EdU incorporation and imaging to assess cell proliferation. Two sample *t*-test was used to compare means of any two groups’ Cyclin D expression, confluency and EdU incorporation in figure **B**, **G**, and **H**. *p < 0.05 and *n* = 3 unless otherwise stated throughout panels.

We next characterized the relationship between gene copy number variations (CNVs) and baseline protein expression of CDK4 pathway nodes across a genetically diverse panel of human ALM and non-ALM cell lines (Fig. 1A, B, Supplementary Fig. 1C). In agreement with clinical observations, CDK4 pathway nodes (*CDK4*, *CDK6*, *CCND1*, *CDKN2A*, *CDKN2B*) were highly dysregulated across our ALM cell lines. CCND1, a key activator of CDK4 and CDK6, was elevated at the protein level in ALM versus non-ALM models (Fig. 1B). Notably, there was no consistent agreement between the copy number status and protein expression of CDK4, CDK6, or cyclin D1 in our ALM panel. ALM cell lines with *CDK4*, *CDK6*, or *CCND1* copy number amplifications did not robustly display elevated CDK4, CDK6, or cyclin D1 protein expression relative to ALM models with normal gene copy numbers, respectively (Supplementary Fig. 1D–F). Analysis of the TCGA to understand the relationship between CNV status, mRNA level, and protein expression for cyclin D1 in patients (*n* = 89 with RPPA information available) with superficial spreading melanoma (CDK4 and CDK6 protein expression unavailable) also revealed no correlation between gene copy number or mRNA expression with protein expression (Supplementary Fig. 1G). This may serve as a cautionary note for the identification of ALM patients who may benefit from CDK4i/6i based solely on tumor sequencing, which may prevent patients with elevated CDK4/CDK6 protein expression but no clear evidence of copy number variation in CDK4/6 pathway genes from treatment.

In agreement with the literature, treatment with the CDK4i/6i palbociclib, ribociclib, or abemaciclib potently inhibits CDK4/6 substrates (p-Rb, FOXM1), and E2F target proteins (PLK1, cyclin A) in a dose-dependent manner (Fig. 1C, Supplementary Fig. 1H, I). Despite potent CDK4/6 and E2F suppression, CDK4i/6i treatment elicited a predominately cytostatic effect, with a subpopulation of viable ALM cells remaining after short-term treatment over the course of three days in the presence of non-physiologically high concentrations of three clinically utilized CDK4i/6i’s palbociclib (>230 nM [10]) (Fig. 1D), ribociclib (>1 mM [11]) (Fig. 1E) and abemaciclib (>290 nM [12]) (Fig. 1F). When the treatment period is extended, therapy-resistant colonies continue to survive following >3-weeks of chronic exposure to CDK4i/6i (Fig. 1G). Accordingly, CDK4i/6i elicited an initial inhibition of proliferative capacity followed by reignition of cell cycle progression following long-term treatment as seen by EdU incorporation (Fig. 1H).

CDK6 protein expression has been recently demonstrated to indirectly predict for sensitivity to CDK4/6 inhibition in ER^+^ breast cancers, non-small cell lung carcinomas, colorectal carcinomas, and superficial spreading melanomas [13]. In contrast, an analysis of correlations for ALM sensitivity to CDK4i/6i suggest that CDK6 protein expression trends (*p* = 0.087) directly with palbociclib sensitivity in ALM (Supplementary Fig. 1J). The baseline protein expression of CDK4 (*p* = 0.72) and Cyclin D1 (*p* = 0.6) in therapy naïve cells did not correlate with ALM sensitivity to CDK4i/6i (Supplementary Fig. 1K, L). Alongside the first phase II clinical trial results of CDK4i/6i treatment in patients with advanced ALM, it was proposed following an analysis of 4 ALM patients that experienced clinical benefit and 5 ALM patients that did not experience clinical benefit to CDK4i/6i that low MCM7 expression and *SH2B3* amplification could serve as predictive biomarkers of poor response to CDK4i/6i [8]. We did not observe significant relationships between MCM7 expression nor SH2B3 expression with sensitivity to any of the three clinically available CDK4i/6i tested (Supplementary Fig. 2A–C). We next put into context our CDK4i/6i sensitivity data with recent reports of potential oncogenic drivers of ALM. LZTR1, an adaptor for Cullin 3 ubiquitin ligase complexes, was proposed to serve as a driver of ALM aggressiveness [9]. No significant relationship emerged between LZTR1 expression and CDK4i/6i sensitivity in our data (Supplementary Fig. 2D). Further, CRKL, a signaling adaptor protein in pathways including the IGF1R-PI3K axis, was proposed to serve as an oncogenic driver of ALM [14]. We also did not observe a significant correlation between *CRKL* expression and CDK4i/6i sensitivity (Supplementary Fig. 2E). Altogether, these data suggest that baseline protein expression of CDK4, CDK6, and cyclin D1 do not correlate with the respective gene copy number status, and the sensitivity of therapy naive ALM cells to single-agent CDK4i/6i does not correlate to: (a) *CDK4*, *CDK6*, or *CCND1* amplification or baseline protein expression, (b) reported CDK4i/6i sensitivity biomarkers (MCM7, SH2B3), or (c) proposed ALM oncogenic drivers (CRKL, LZTR1).

### Loss of DUSP4 expression following CDK4i/6i promotes ERK activation and drives intrinsic resistance via cyclin D1

**Fig. 2:**
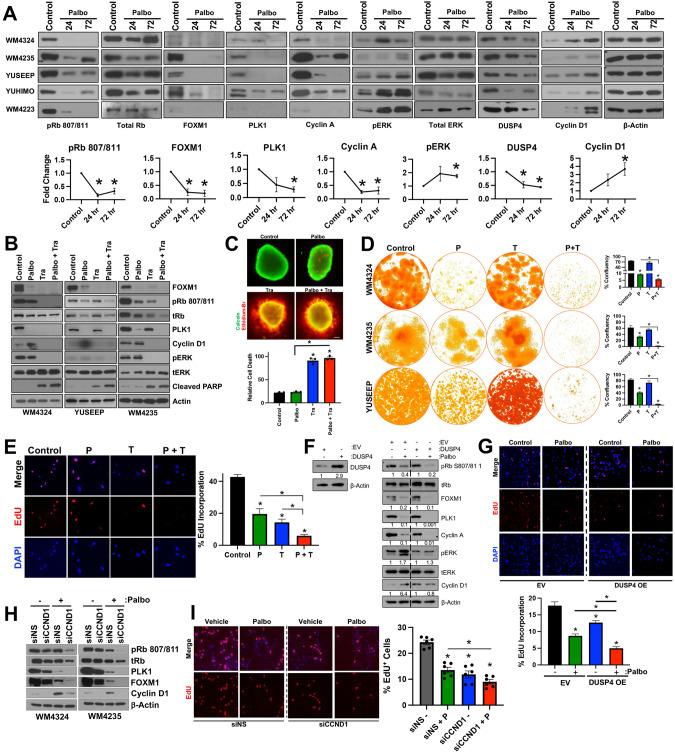
ERK hyperactivation drives intrinsic CDK4i/6i resistance via cyclin D1. **A** A panel of ALM cell lines were treated with palbociclib (500 nM, 24–72 h) before characterization by Western blotting (top panel). Densitometric analysis is shown in the bottom panel (*n* = 5 in each group). **B** Cells were treated with palbociclib (500 nM) and/or trametinib (10 nM for WM4324/WM4235, 1 nM for YUSEEP) for 72 h before characterization by Western blotting. **C** WM4324 spheroids were formed before implantation in collagen and treatment with palbociclib and/or trametinib for 72 h. Spheroids were subsequently stained with a viability stain and imaged by fluorescent microscopy (green indicates living cells, red indicates dead cells). **D** Cells were treated for up to 4 weeks with palbociclib (30 nM) and/or trametinib (0.3 nM) before colonies were fixed and stained with crystal violet. Quantification is shown in the right panel. **E** WM4235 cells were treated with palbociclib (500 nM) and/or trametinib (10 nM) for 24 h before subsequent staining with EdU. Shown in the panel to the right is quantification. **F** YUHIMO cells were stably transfected with either Empty Vector (EV) or DUSP4 (DUSP4 OE) in the presence or absence of palbociclib (500 nM) for 72 h before characterization by Western blotting. **G** YUHIMO cells were transfected with either EV or DUSP4 for 72 h before treatment with palbociclib (500 nM) for 72 h before proliferative capacity following EdU staining. **H** Cells were transfected with either non-specific siRNA (siNS) or siCCND1 in the presence or absence of palbociclib (500 nM) for 72 h before characterization by Western blotting. **I** WM4324 cells were transfected with either non-specific siNS or siCCND1 for 48 h before treatment with palbociclib (500 nM) for 6 h before proliferative capacity following EdU staining (*n* = 6 in each group). Paired *t*-test was used to calculate the significance of difference between treatment and control group in figure **A**. Ordinary One-Way ANOVA and Tukey test were used to perform multiple pairwise comparisons in figure **C**, **E**, and **G**. Two sample *t*-test was used to compare group means in figure **D** and **I**. *p < 0.05 and *n* = 3 unless otherwise stated throughout panels.

Improvement in the efficacy of CDK4i/6i with inhibitors of the MAPK pathway has been reported in prostate adenocarcinoma [15], superficial spreading melanoma [16, 17], and uveal melanoma [18]. In the context of *NRAS* mutant superficial spreading melanoma, network modeling of tumor cells treated with MEKi has identified CDK4 as a key driver of therapy resistance [19]. However, the mechanistic connection between CDK4/6 activity and MAPK pathway signaling remains incompletely understood. We next investigated the MAPK pathway following CDK4i/6i in our ALM system and observe that although acute palbociclib treatment led to reduced activity of downstream CDK4/6 substrates (pRb, FOXM1) and E2F effectors (PLK1, cyclin A), a robust hyperactivation of MAPK signaling (pERK) and increased downstream cyclin D1 expression were observed across our ALM panel irrespective of *NRAS* or *BRAF* mutational or CNV status (Fig. 2A, Supplementary Fig. 1C). For example, CDK4i/6i-induced activation of ERK occurred in *BRAF*^V600E^ mutant (WM4324), *NRAS*^Q61R^ mutant (WM4235), as well as *BRAF*/*NRAS* wildtype (YUSEEP) cells. Further, we observe equivalent hyperactivation of pERK and elevation of cyclin D following CDK4i/6i in AM cell lines with (a) wild type *BRAF/NRAS* copy numbers (WM4223), (b) *NRAS* copy number deletion (YUHIMO), (c) *NRAS* copy number gains (YUSEEP, WM4235), and (d) *BRAF* copy number gains (WM4324), which suggests that what we have observed cannot be solely explained by elevated NRAS/BRAF signaling given the heterogeneity of BRAF/NRAS mutational and CNV status across our panel. Hyperactivation of ERK and increased cyclin D1 expression were also observed following pharmacological inhibition of CDK4/6 with abemaciclib, ribociclib and genetic silencing of CDK4/6 (Supplementary Fig. 3A, B).

We next tested whether targeting the MAPK pathway with a MEK1/2 inhibitor (MEKi, trametinib) could ablate intrinsic CDK4i/6i resistance. Combination treatment with MEKi and CDK4i/6i decreases cell cycle proteins (FOXM1, p-Rb, cyclin D1, PLK1) to a greater extent than what was achievable by either compound as a single-agent (Fig. 2B), and increases apoptosis (cleaved PARP) relative to single-agent CDK4i/6i. Targeting the MAPK pathway at the level of ERK (VX-11e) also increased the capacity of CDK4i/6i to decrease cell cycle proteins (Supplementary Fig. 3C). Combination treatment with MEKi and CDK4i/6i increased the 3D cytotoxicity in ALM spheroids (Fig. 2C) relative to single-agent CDK4i/6i treatment alone. Further, concurrent MEKi + CDK4i/6i conferred the greatest antitumor durability in long-term colony formation assays (Fig. 2D), and most efficiently reduced the subpopulation of EdU^+^ ALM cells relative to single-agent treatment alone (Fig. 2E).

It was previously reported that reactivation of the MAPK pathway following BRAFi in *BRAF*^V600E^ mutant superficial spreading melanomas was driven, in part, by reduced expression of proteins that negatively regulate the pathway, including members of the Sprouty (SPRY) dual specificity phosphatase (DUSP) family [20]. In our ALM cell line panel, we observe that CDK4i/6i treatment decreases protein expression of DUSP4 levels, not SPRY2 or DUSP6 (Fig. 2A, Supplementary Fig. 3D). We next tested the hypothesis that alterations in DUSP4 protein levels could be contributing to the hyperactivation of the MAPK pathway following CDK4i/6i. Overexpression of DUSP4 ablates the activation of ERK and induction of cyclin D1 expression following CDK4i/6i (Fig. 2F), overexpression of DUSP4 increases the antiproliferative efficacy of CDK4i/6i, as evidenced by the greatest reduction of cell cycle machinery and improved inhibition of EdU positivity relative to CDK4i/6i treatment alone (Fig. 2G), and overexpression of DUSP4 increases the ability of CDK4i/6i to suppress clonogenic outgrowth in long-term colony formation assays (Supplementary Fig. 3E). In contrast, genetic silencing of DUSP4 reduced the efficacy of CDK4i/6i (Supplementary Fig. 3F). Altogether, these results demonstrate the role CDK4i/6i-induced reduction of DUSP4 serves in ALM cell sensitivity to CDK4i/6i.

The MAPK pathway has been experimentally shown to regulate cyclin D1 in melanocytes and *BRAF*^V600E^ superficial spreading melanoma [21], however, the connection between MAPK activity and cyclin D1 expression has not yet been established in ALM. Growing ALM cells in nutrient-replete media following serum starvation induces MAPK activity (pERK, pRSK) and downstream cyclin D1 expression, which could be blocked using MEKi or ERKi (VX-11e), demonstrating the MAPK pathway, at least in part, regulates cyclin D1 expression in ALM (Supplementary Fig. 3G). We next tested the hypothesis that ERK hyperactivation following CDK4i/6i preserves cellular proliferation by promoting cyclin D1 expression. Genetic silencing of cyclin D1 increased the cell cycle arrest potential of CDK4i/6i, evidenced by further decreased protein expression of pRb, PLK1, and FOXM1 (Fig. 2H). Genetic silencing of cyclin D1 also decreased the EdU^+^ subpopulation relative to what CDK4i/6i alone could achieve (Fig. 2I). In summary, these data suggest ALM cells adaptively escape single-agent CDK4i/6i by hyperactivating the MAPK pathway via, at least in part, reduced DUSP4 protein expression. The hyperactivation of ERK activity maintains the proliferative capacity of ALM cells treated with CDK4i/6i by promoting cyclin D1 expression.

### ERK hyperactivation drives acquired resistance to CDK4i/6i

**Fig. 3:**
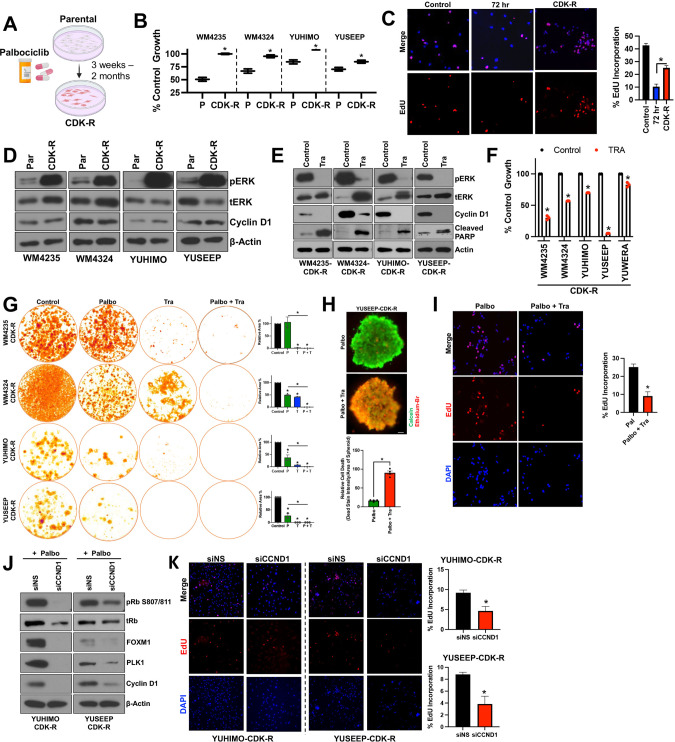
ALMs acquire resistance to CDK4i/6i via ERK hyperactivation. **A** ALM cell lines were treated with increasing concentrations of palbociclib (50–500 nM; up to 2 months) to generate cells with acquired resistance (CDK-R). **B** Parental and CDK-R cells were treated with palbociclib (100 nM; 72 h) before cell number was quantified by MTT. **C** Proliferative capacity was assessed in WM4235 cells treated with palbociclib (500 nM; 72 h) and WM4235-CDK-R by EdU staining. Quantitation is shown in the right panel. **D** Parental and CDK-R pairs were characterized by Western blotting. **E** CDK-R cells were treated with trametinib (10 nM; 24 h) while in the constant presence of palbociclib (500 nM) before Western blotting characterization. **F** CDK-R cells were treated with trametinib (10 nM; 72 h) before cell number was quantified by MTT. **G** CDK-R cells were treated with palbociclib (500 nM) and/or trametinib (1 nM) for up to 4 weeks before colonies were fixed and stained with crystal violet. Quantification is shown in the lower panel. **H** YUSEEP-CDK-R spheroids were generated, implanted in collagen, and treated with palbociclib +/− trametinib for 72 h before viability staining and imaging (*n* = 4 for each condition). **I** WM4235-CDK-R cells were treated with palbociclib (500 nM) and/or trametinib (10 nM) for 24 h before staining with EdU. Quantitation is shown in the bottom left panel (*n* = 4 for each condition). **J** Cells were treated with siCCND1 in the presence of palbociclib (500 nM) for protein lysate was immunoblotted. **K** Cells were treated as in (**J**) before staining with EdU. Two sample *t*-test was used for pairwise comparisons throughout panels. *p < 0.05 and *n* = 3 unless otherwise stated.

We next investigated the role of the MAPK pathway in acquired resistance to CDK4i/6i in ALMs. We generated ALM models with acquired CDK4i/6i-resistance (CDK-R) by treating therapy naïve ALM cell lines with increasing concentrations of palbociclib (10–500 nM) between 3 weeks and 2 months (Fig. 3A). CDK-R cells display reduced sensitivity to acute treatment with palbociclib (Fig. 3B), cross-resistance to ribociclib (Supplementary Fig. 4A), and regain their proliferative (EdU^+^ positivity) capacity (Fig. 3C). Notably, CDK-R cells display elevated phospho-ERK relative to parental cells (Fig. 3D), which functionally drives CDK4i/6i resistance as evidenced by induction of PARP-1 cleavage, reduction in cell cycle proteins, and decreased viability following combination treatment with MEKi (Fig. 3E, F). CDK-R also exhibited decreased sensitivity to CDK4i/6i in long-term colony formation assays relative to their respective parental cell lines (Fig. 3G, Supplementary Fig. 4B). Treatment with MEKi resensitized CDK-R cells to long-term treatment with CDK4i/6i (Fig. 3G), induced cytotoxicity in 3D CDK-R spheroids (Fig. 3H) and depleted the proliferative EdU^+^ subpopulation of CDK-R cells (Fig. 3I). Treatment of CDK-R cells with ERKi also induced significant suppression of cell cycle machinery (e.g., PLK1, FOXM1, cyclin D1) and induced apoptosis (e.g., PARP cleavage) (Supplementary Fig. 4C). Of note, we observed cell growth in select CDK-R cell lines was still affected by CDK4i/6i, which would suggest the existence of reversible mechanisms of acquired resistance to palbociclib that have been previously reported in cholangiocarcinoma cells [22].

Activation of ERK in the context of acquired CDK4i/6i-resistance has not been reported in melanoma, however evidence in the breast cancer literature proposes a role for de novo *HER2* mutations in estrogen receptor positive (ER^+^) breast cancers [23]. We sequenced for *HER2* mutations and copy number variations (CNVs) in our CDK-R models with acquired CDK4i/6i resistance versus their respective therapy naïve parental, and observe no new *HER2* mutations or copy number gains (Supplementary Fig. 4D). Heterozygous *RB1* loss was also recently reported as a biomarker for CDK4i/6i resistance in ER^+^ breast cancer. We observe *RB1* deletion in one of our CDK-R models (YUHIMO-CDK-R), but no evidence of *RB1* inactivating mutations across our CDK-R models relative to their respective parentals. In addition, evidence in hormone receptor positive (HR^+^) breast cancers suggest potential roles for upstream receptor overexpression (FGFR2) and de novo mutations in RAS, AKT1, AURKA, CCNE2, and/or ERBB2 in the activation of ERK following acquired CDK4i/6i resistance [24]. We sequenced for *AKT1*, *FGFR2*, *FGFR3*, *FGFR4*, and *NRAS* mutations and CNVs in our CDK-R models versus their respective therapy naïve parental counterparts, and observe no new mutations in these genes. We observed a copy number gain of *NRAS* in the WM4324 CDK-R cells versus its respective parental. No other new copy number gains were observed (Supplementary Fig. 4D).

In contrast to what we observed in therapy naïve cells treated with CDK4i/6i, three out of the four CDK-R cell lines expressed similar cyclin D1 levels relative to their respective parentals (Fig. 3D), which suggest rewiring of cyclin D1 expression may occur over time in the context of chronic treatment. However, genetic silencing of cyclin D1 also resensitized CDK-R cells to palbociclib as evidenced by reduced expression of pRb, FOXM1, and PLK1 (Fig. 3J) and depletion of EdU+ cells (Fig. 3K). Altogether, these results indicate that hyperactivation of the MAPK pathway drives acquired CDK4/6 inhibitor resistance and targeting MEK1/2 or cyclin D1 can reinforce CDK4i/6i efficacy.

### MEKi increases the in vivo efficacy of CDK4i/6i in therapy naïve and acquired CDK4i/6i-resistant ALM cells

**Fig. 4:**
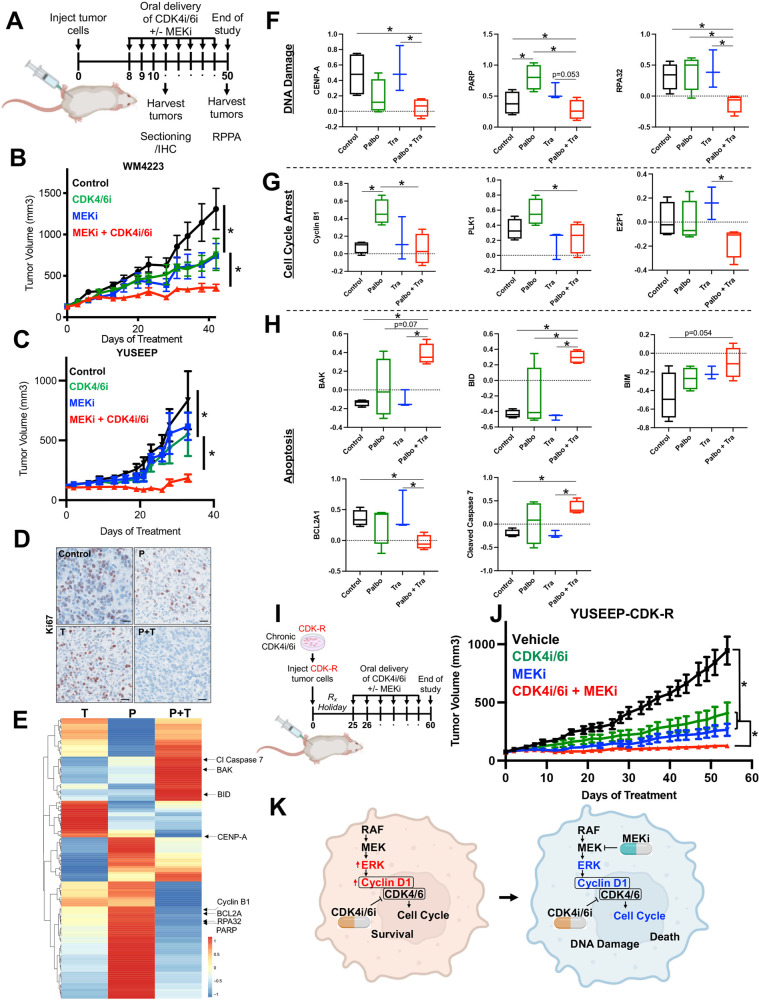
MEKi increases the in vivo efficacy of CDK4i/6i in therapy naïve and acquired CDK4i/6i-resistant ALM models. **A** Schematic detailing the therapy naïve trial strategy. **B** Tumor growth curves of NSG mice implanted with the ALM PDX WM4223 and treated with vehicle control, palbociclib, trametinib, or the combination of palbociclib plus trametinib via oral gavage. **C** Tumor growth curves of NSG mice implanted with YUSEEP cells and treated with vehicle control, palbociclib, trametinib, or the combination of palbociclib plus trametinib via oral gavage. **D** IHC staining for Ki67 in WM4223 PDX tumor tissue from mice treated for 3 days with vehicle control, palbociclib, trametinib, or the combination of palbociclib + trametinib. **E** Heatmap of significant differentially expressed proteins between the palbociclib and control arm, the trametinib and control arm, and the combination palbociclib plus trametinib and control arm. **F** Plots depicting differentially expressed DNA repair and damage proteins from the data in (**E**), (**G**) Plots depicting differentially expressed cell cycle proteins from the data in (**E**), (**H**) Plots depicting differentially expressed apoptosis proteins from the data in (**E**). **I** Schematic detailing the acquired CDK4i/6i-resistant trial strategy. **J** Tumor growth curves of NSG mice implanted with the YUSEEP-CDK-R cells and treated with palbociclib, trametinib, or the combination of palbociclib plus trametinib via oral gavage. **K** Graphic summary of the manuscript findings. Wilcoxon signed-rank test was used to calculate significance when comparing tumor growth curves in figure **B**, **C** and **G**, 13, 12 and 24 time points are involved respectively, and *n* = 3 for each time point. Two sample *t*-test was used in figure **F**, **G** and **H** (*n* = 4 for control, *n* = 4 for Palbo, *n* = 3 for Trametinib, *n* = 4 for Palbo + Trametinib). *p < 0.05 unless otherwise stated.

To assess the utility of targeting MEK to increase the in vivo efficacy of CDK4i/6i, we implanted the WM4223 patient-derived xenograft (PDX) model, derived from a biopsy of a metastatic ALM that originated in the left foot of a 73 year old male patient, into NOD.Cg-*Prkdc*^*scid*^ *Il2rg*^*tm1Wjl*^/SzJ (NSG) mice (Fig. 4A). After 1–2 weeks, tumors were palpable and mice were treated via oral gavage with vehicle control, palbociclib (25 mg/kg), trametinib (0.3 mg/kg) or the combination of palbociclib plus trametinib, which was well tolerated (Supplementary Fig. 5A). Palbociclib and trametinib each conferred significant anti-tumor activity as single-agent treatments; however, the greatest therapeutic benefit was observed in mice receiving combination palbociclib plus trametinib treatment (Fig. 4B, Supplementary Fig. 5B). The ALM cell line YUSEEP, derived from the left heel, was also implanted in NSG mice and treated with vehicle control, palbociclib, trametinib, of the combination of palbociclib plus trametinib once tumors were palpable. Combination palbociclib plus trametinib treatment again conferred significantly greater antitumor activity relative to what could be accomplished by the single-agents alone (Fig. 4C, Supplementary Fig. 5C, D). Concurrent treatment with palbociclib and trametinib resulted in the greatest inhibition of cell cycle machinery (i.e., pRb, FOXM1, PLK1, cyclin D1) in lysate collected from a subset of tumor-bearing mice sacrificed after 3 days of treatment (Supplementary Fig. 5E) and conferred the greatest decrease of Ki67 staining in tumor tissue (Fig. 4D). At treatment endpoint for the WM4223 in vivo study (day 50), tumor tissue was characterized by reverse-phase protein array (RPPA) to identify the mechanism(s) of action underlying the long-term therapeutic efficacy of MEKi + CDK4i/6i relative to single-agent therapy (Fig. 4E). A total of 46 proteins were significantly differentially expressed between vehicle control tumors and combination palbociclib plus trametinib treated tumors that were not observed in the single-agent treated tumors (Supplementary Table). Interestingly, a signature indicative of reduced DNA repair capacity (decreased CENP-A, PARP and RPA32 protein expression) correlated with increased double strand DNA breaks in tumors treated with combination palbociclib plus trametinib (Fig. 4F, Supplementary Fig. 5F). Combination palbociclib plus trametinib treatment also resulted in the greatest cell cycle arrest signature (as seen by decreased cyclin B1, PLK1 and E2F1 protein expression (Fig. 4G)), and most significant induction of apoptosis (increased BAK, BID, BIM, caspase 7 cleavage, and reduced BCL2A1 protein expression) (Fig. 4H). In agreement, treatment of ALM cells with combination palbociclib plus trametinib for 10 days in vitro displayed increased DNA damage, BIM expression and cleavage of caspase 7 (Supplementary Fig. 5G).

To assess the utility of targeting MEK to overcome acquired resistance to CDK4i/6i in vivo, YUSEEP-CDK-R cells chronically treated with palbociclib in vitro were implanted in NSG mice (Fig. 4I). In line with observations of reversible (non-heritable) mechanisms of acquired resistance to palbociclib [22], YUSEEP-CDK-R cells that expanded in vivo during a >3 week drug holiday again exhibited sensitivity to single-agent palbociclib (Fig. 4J). Although vehicle treated YUSEEP-CDK-R tumors exhibited a greater growth rate relative to palbociclib treated, the vehicle treated YUSEEP-CDK-R tumors grew slower than the vehicle treated parental YUSEEP tumors (Fig. 4C). Treatment with MEKi significantly blunted tumor growth of CDK4i/6i-resistant ALM cells, and the combination of CDK4i/6i + MEKi resulted in the greatest antitumor activity and decrease in cell cycle proteins (pRb, cyclin D, PLK1, FOXM1) relative to what could be achieved by the single-agents alone (Fig. 4J, Supplementary Figure H, I, J). These findings indicate that continuous pressure from CDK4i/6i is required to maintain maximal vulnerability to MEKi. Altogether, these results underscore the importance of the MAPK pathway in driving intrinsic and acquired CDK4i/6i resistance in ALM and the translational potential of MEKi to increase the in vivo antitumor activity of CDK4i/6i against therapy naïve and CDK4i/6i-resistant ALMs, via increased DNA damage, cell arrest and tumor cell death (Fig. 4K).

## Discussion

ALM represents a distinct disease from other forms of CM and the therapy resistance landscape that limits the curability of patients with advanced ALM is poorly understood. ALM remains the most lethal form of CM and existing therapies effective in other forms of CM (e.g., BRAFi, ICB) are not as active in ALM for reasons that are poorly understood. Genetic alterations in the CDK4-pathway occur in the majority of ALM cases, and there is preclinical evidence that ALM cells with CDK pathway alterations are highly sensitive to CDK4i/6i [7]. Unfortunately, these findings have not translated clinically, with single agent palbociclib conferring a mPFS of less than 3 months suggesting mechanisms of resistance blunt efficacy. Enthusiasm for the clinical testing of CDK4i/6i-containing therapy strategies in patients with other forms of melanoma has grown in the past decade stemming from (a) observations of CDK4-pathway alterations in >90% of CM cases, (b) evidence of downstream activation of CDK4/6 as a consequence of elevated MAPK pathway signaling, and (c) a role for CDK4 in ICB resistance [25]. Clinical trials have commenced incorporating CDK4i/6i plus MEKi in *NRAS* mutant CM [19, 26], CDK4i/6i plus BRAFi/MEKi in *BRAF* mutant CM [27], and most recently CDK4i/6i plus immune checkpoint blockade [28]. Mechanisms of intrinsic resistance to CDK4i/6i have been reported in other forms of melanoma, but no studies have reported CDK4i/6i resistance mechanisms in ALM models.

Here, we report a seminal investigation into the underlying intrinsic and acquired resistance mechanisms that blunt the therapeutic efficacy of CDK4i/6i in ALM. Our study finds that despite the robust inhibition of CDK4/6 substrates and E2F1 effector proteins by single-agent CDK4i/6i, most tumor cells remain viable following treatment with non-physiologically high concentrations of CDK4i/6i that results in a temporary cytostatic response in vitro and in vivo followed by reactivation of the cell cycle. A recent study proposed a role for elevated CDK6 expression in the intrinsic resistance of NSCLC and superficial spreading melanomas to CDK4i/6i, however, we find the opposite in ALM models whereby CDK6 expression directly trends with CDK4i/6i efficacy. It has been proposed that ALM models with CDK4 pathway alterations (defined as *CDK4* gain, *CCND1* gain, *CDKN2A* loss) exhibit elevated sensitivity to CDK4i/6i [7], however, we could not corroborate a differential sensitivity between ALM models based off the status of the CDK4 pathway. This lack of robust correlation was also reported during the first phase II clinical trial of palbociclib in ALM patients, whereby the authors concluded “neither the genetic status nor the protein expression level of CDK4, CCND1, or CDKN2A was significantly associated with clinical response to palbociclib” [8]. Further, we find a notable discordance between the genetic status of the CDK4 pathway and the protein expression of key nodes (e.g., CDK4, CDK6), suggesting both genetic and proteomic characterization of the CDK4 pathway should be performed when stratifying patients for treatment with CDK4i/6i. We are cognizant that the number of cell lines here limit our statistical power to draw definitive conclusions, but these studies provide additional evidence for hypothesis generation. Future studies with expanded numbers of ALM cell lines will contribute to determining whether these biomarkers have utility in patient stratification for clinical trial inclusion.

We examined the role of MAPK pathway signaling in the context of intrinsic and acquired CDK4i/6i resistance, in part, due to evidence of possible synergies of combination CDK4i/6i plus MAPK pathway inhibitor treatment in breast cancer and superficial spreading melanomas with either *NRAS* or *BRAF* mutations. Our investigation identified a rapid hyperactivation of the MAPK pathway occurs within three days of CDK4i/6i treatment in the context of therapy naïve ALM, which drives downstream expression of cyclin D1. The MAPK pathway hyperactivation and increase in cyclin D1 expression each functionally drove intrinsic CDK4i/6i resistance, which could be reversed by either incorporating a clinically relevant MEK inhibitor (trametinib), an ERK inhibitor (VX-11e), or genetic silencing of cyclin D1. Of note, evidence of MAPK pathway hyperactivation following acute (0–72 h) CDK4i/6i treatment has not been reported previously in ALM or other melanoma subtypes. Although MAPK hyperactivation has been reported in other cancer types including head and neck [29] and luminal A breast [30] following acute CDK4i/6i, the underlying mechanism remains poorly understood. We here demonstrate that CDK4i/6i results in decreased protein expression of the negative regulator of ERK activity, DUSP4. Loss of DUSP4 is functional in the CDK4i/6i-induced activation of ERK, as DUSP4 overexpression ablates CDK4i/6i-induced ERK activation, increases the anti-proliferative activity of CDK4i/6i, and reinforces the capacity of CDK4i/6i to suppress clonogenic outgrowth in the context of chronic treatment. These results are in line with a previous report in ER^+^ breast cancer where genetic silencing of CCND1 increased the antiproliferative capacity of combination treatment with fulvestrant and CDK4i/6i [31].

In the context of acquired resistance, a robust hyperactivation of the MAPK occurs across our panel of CDK-R cells relative to their respective parental lines. Elevated MAPK activity functionally drove resistance in CDK-R cells and could be reversed with the use a MEKi or ERKi. Prior evidence in ER^+^ breast cancer suggests de novo *HER2* mutations can hyperactivate ERK activity in the context of acquired CDK4i/6i resistance. We confirmed in our paired parental and CDK-R ALM models the lack of de novo *HER2* mutations following acquired CDK4i/6i resistance. Further, it has been observed by others that overexpression of FGFR2 and/or de novo mutations in *RAS*, *AKT1*, *AURKA*, *CCNE*, and *ERBB2* could possibly contribute to MAPK activation following acquired CDK4i/6i resistance. We sequenced for *AKT1*, *FGFR2*, and *NRAS* mutations and CNVs in our CDK-R models with acquired CDK4i/6i resistance versus their respective therapy naïve parental line, and observe no new mutations in *AKT1*, *FGFR2*, or *NRAS* across our CDK-R and parental cell line pairs. We did observe a copy number gain of *NRAS* in the WM4324 CDK-R cell versus its respective parental cell line. Outside activation of the MAPK pathway, *RB1* loss and *FAT1* loss were recently reported to contribute to CDK4i/6i resistance in ER^+^ breast cancers [32]. We observe *RB1* deletion also occurs in one of the three CDK-R/parental ALM cell line pairs interrogated. In ER^+^ breast cancer models, PI3K-dependent intrinsic resistance has been observed [33], which should also be investigated in future studies in the context of ALM.

In contrast to the induction of cyclin D1 observed in all therapy naïve ALM cell lines treated with CDK4i/6i for 24–72 h, there was no change in cyclin D1 protein expression in three out of the four CDK-R cell lines relative to their respective parental lines, suggesting rewiring of cyclin D1 expression may occur in the context of chronic drug exposure. Nonetheless, genetic silencing experiments reveal CDK-R cells, at least in part, rely on cyclin D1 for proliferation. In summary, our findings define MAPK pathway plasticity as an underlying mechanism of intrinsic and acquired CDK4i/6i therapy resistance in ALM. Further, this body of work makes numerous clinically relevant observations for the treatment of patients with advanced ALM including that (a) *CDK4* and *P16INK4A* status does not robustly predict ALM patient survival, (b) *CDK4* pathway alterations do not robustly predict ALM sensitivity to CDK4i/6i, (c) CDK6 protein expression does not robustly predict ALM sensitivity to CDK4i/6i, and (d) genetic status (e.g., copy number variation) does not correlate with protein expression of CDK4 pathway nodes. These findings provide the rationale to further investigate combination treatment of CDK4i/6i plus MEKi as first- and second-line therapy in patients with advanced ALM.

## Methods

### Cell culture and reagents

Melanoma cell lines YUSEEP, YUHIMO, YUWERA, and YUCRATE were obtained from Ruth Halaban (Yale University) in 2020. Melanoma cell lines WM4235, WM4324, WM9, 1205Lu, WM989, WM983B, WM4380 and WM4258, as well as the PDX model WM4223 were obtained from Meenhard Herlyn (Wistar Institute) in 2020. All patient samples were collected under institutional review board (IRB) approval [34]. Cell lines were tested for Mycoplasma biannually and authenticated using short-tandem repeat fingerprinting. All cell lines are cultured in RPMI-1640 (Corning,10-040-CM) supplemented with 5% fetal bovine serum (FBS; Cytiva, SH30109.03) in the presence of 5% CO_2_ at 37 °C. Commercially purchased compounds include palbociclib (SelleckChem, S1116), ribociclib (SelleckChem, S7440), abemaciclib (Apex Biotechnology, A1794), trametinib (SelleckChem, S2673), AZD6244 (SelleckChem, S1008) and VX-11e (SelleckChem, S7709).

### siRNA transfection and overexpression

Cells were transfected for 24 h with siNS and siCCND1 (Dharmacon, D-001810-10-50) at a final concentration of 20 nMol/L using Lipofectamine 2000 (Invitrogen, 11668-019) transfection reagent. Cells were harvested after 72 h of knockdown before characterization by immunoblotting. YUHIMO cells were transduced with the virus generated from the DUSP4 plasmid (VectorBuilder, VB900124-1623hnw) and were selected with Puromycin (0.5 ug/ml) to generate stables cell lines.

### Immunoblotting, cell cycle analysis, EdU staining, and fluorescent microscopy

Protein lysates were immunoblotted as previously described [35] with the following antibodies CDK4, CDK6, total Rb, phospho-Rb Ser807/811, PLK1, Cyclin A, Cyclin B1, Cyclin D1, β-Actin, FOXM1, PTEN, p16, phospho-ERK1/2 Thr202/204, total ERK1/2, and cleaved Parp from Cell Signaling Technology. Densitometric analysis was performed by utilizing the Gels function in ImageJ. Individual gel lanes were identified and manually outlined. The band intensity of each gel lane was then plotted by the ImageJ software and the subsequent peaks created were used to quantify the relative protein quantity. The software automatically calculates this based on the area under each peak. The results were then normalized to the protein quantity of β-Actin and control lanes. Fluorescent microscopy was performed as previously described [35] using the manufacturer’s instructions from the EdU kit (Click-iT^TM^ EdU Alexa Fluor^TM^ 647 Imaging Kit, C10340. For cell cycle analysis, cells were plated at 1 × 10^5^ per well in a 6-well plated and treated, as indicated. Floating and adherent cells were pooled, pelleted, washed with cold PBS and fixed with 70% ethanol. Fixed cells were subsequently washed, resuspended in PBS, treated with RNase A solution and stained with propidium iodide (0.5 mg/mL, BioLegend, 421301) Cell cycle analysis was performed on Cytek^TM^ NL-3000 and data were analyzed using FlowJo Software.

### Cell viability MTT assay and clonogenic assay

For MTT assays, cells were at 2,000/well in 96-well plates and treated as indicated for 72 h before thiazolyl blue tetrazolium bromide was added to growth medium, incubated for 4 h at 37 °C, solubilized and color was quantified on a 96 well plate reader (Synergy H1 microplate reader; BioTek) at the absorbance 570 nm. For clonogenic assays, cells were plated at 2–5 × 10^3^ per well in a 6-well plate and treated twice a week for up to 4 weeks as indicated before colonies were stained with crystal violet. Plates were imaged and quantified by the Colony Area ImageJ plug-in. Individual wells were cropped by the software and thresholds were created automatically to remove the background. Manual cropping and thresholding was performed when image artifacts compromised the software’s ability to properly identify the background. The Colony Measurer function was then used to quantify the percent area covered by cell colonies in each thresholded well.

### Massively parallel sequencing

DNA from cell lines (WM4235, WM4324, YUHIMO, YUCRATE, YUSEEP, YUWERA) was characterized by massively parallel sequencing using a custom-designed, targeted panel as previously described [34, 36]. Mutational information for WM4223 was derived from previous targeted sequencing.

### Reverse-phase protein arrays

Proteins were isolated from tumor shears and cell lines, and RPPA analysis was performed as previously described [37]. Prior antibody testing confirmed the specificity of each antibody, and direct correlation between RPPA and Western blotting results (data not shown). A logarithmic value was generated, reflecting the quantitation of the relative amount of each protein in each sample. Differences in relative protein loading were determined by the median protein expression for each sample across all measured proteins using data that had been normalized to the median value of each protein. The raw data were then divided by the relative-loading factor to determine load-corrected values. Logarithmic values for each protein were mean-centered to facilitate concurrent comparisons of different proteins [37].

### RNA sequencing

RNA Sequencing for baseline levels of YUCRATE, YUSEEP, YUHIMO, YUWERA, WM4324, WM4235 was conducted at GENEWIZ, LLC./Azenta US, Inc (South Plainfield, NJ, USA). Total RNA samples were quantified using Qubit 2.0 Fluorometer (Life Technologies, Carlsbad, CA, USA) and RNA integrity was checked with 4200 TapeStation (Agilent Technologies, PaloAlto, CA, USA). RNA samples were initially treated with TURBO DNase (Thermo Fisher Scientific, Waltham, MA, USA) to remove DNA contaminants. RNA sequencing libraries were prepared using the NEBNext Ultra Directional RNA Library Prep Kit for Illumina following manufacturer’s instructions (NEB, Ipswich, MA, USA). The sequencing libraries were multiplexed and clustered on three lanes of a flowcell. After clustering, the flowcell was loaded on the Illumina HiSeq 4000 instrument according to manufacturer’s instructions. The samples were sequenced using a 2 × 150 Pair-End (PE) configuration.

### In vivo experiments

Xenograft studies were performed using 6–8 week old NSG mice (Charles River Laboratories) in an Association for the Assessment and Accreditation of Laboratory Animal Care-accredited facility. WM4223 (5 × 10^5^) cells or YUSEEP-CDK-R (3 × 10^5^) cells were implanted subcutaneously into NSG mice and stratified into the indicated treatment arms when tumors were palpable (~150 mm^3^) to begin treatment. Mice were treated with either palbociclib (25 mg/kg, oral gavage), trametinib (0.3 mg/kg oral gavage) or the combination of palbociclib and trametinib. Tumor sizes were measured every 2 days using digital calipers. Tumor volumes were calculated using the following formula: volume = 0.5 x (length × width^2^).

### TCGA correlation test

In the TCGA (The Cancer Genome Atlas) database, there are 89 skin cutaneous melanoma patients with paired open source RPPA cyclin D1 data and CCND1 mRNA data in primary tumor. Pearson correlation test was performed between cyclin D1 protein level and CCND1 mRNA TPM (transcript per million) level across 89 samples. The null hypothesis of the test is that there is no correlation between the two variables. *P* value larger than 0.05 indicates we cannot reject null hypothesis. There are 90 skin cutaneous melanoma patients with paired RPPA cyclin D1 data and copy number variation data of the primary tumor in the TCGA. Similarly, person correlation test was performed between cyclin D1 protein level and CCND1 copy number variation across these 90 patients.

### Statistical analysis

GraphPad Prism 9 statistical software was used to perform Student’s *t* test, Ordinary One-Way ANOVA and Tukey’s multiple comparison test where *indicates *p* < 0.05. Bar plots show the mean of at least 3 independent experiments, with error bar representing standard deviation. Linear mixed models were used to estimate and compare tumor growth rates (mm^3^/day) between treatment groups. R was used to perform Pearson Correlation test and Wilcoxon signed-rank test.

### Supplementary information

Supplemental Figure 1

Supplemental Figure 2

Supplemental Figure 3

Supplemental Figure 4

Supplemental Figure 5

Supplemental Figure Legends

Supplemental Material

## Data Availability

Copy number variation data of relevant genes for baseline Acral and non-Acral cell lines in Fig. 1A, WM4223 tumor median centered protein RPPA data in Fig. 4F, G, H, copy number variation and mutation data of relevant genes for Supplementary Fig. 4D are stored in supplementary data. Raw RNA sequencing data of baseline Acral melanoma cell lines YUCRATE, YUSEEP, YUHIMO, YUWERA, WM4324, WM4235 generated from this study have been deposited in Sequence Read Archive (SRA) under accession PRJNA953970.
