# Supplementary figures and images for: ERK hyperactivation serves as a unified mechanism of escape in intrinsic and acquired CDK4/6 inhibitor resistance in acral lentiginous melanoma
Source: Oncogene. 2023 Dec 8;43(6):395–405. doi: 10.1038/s41388-023-02900-6 (PMC10837073; doi:10.1038/s41388-023-02900-6)

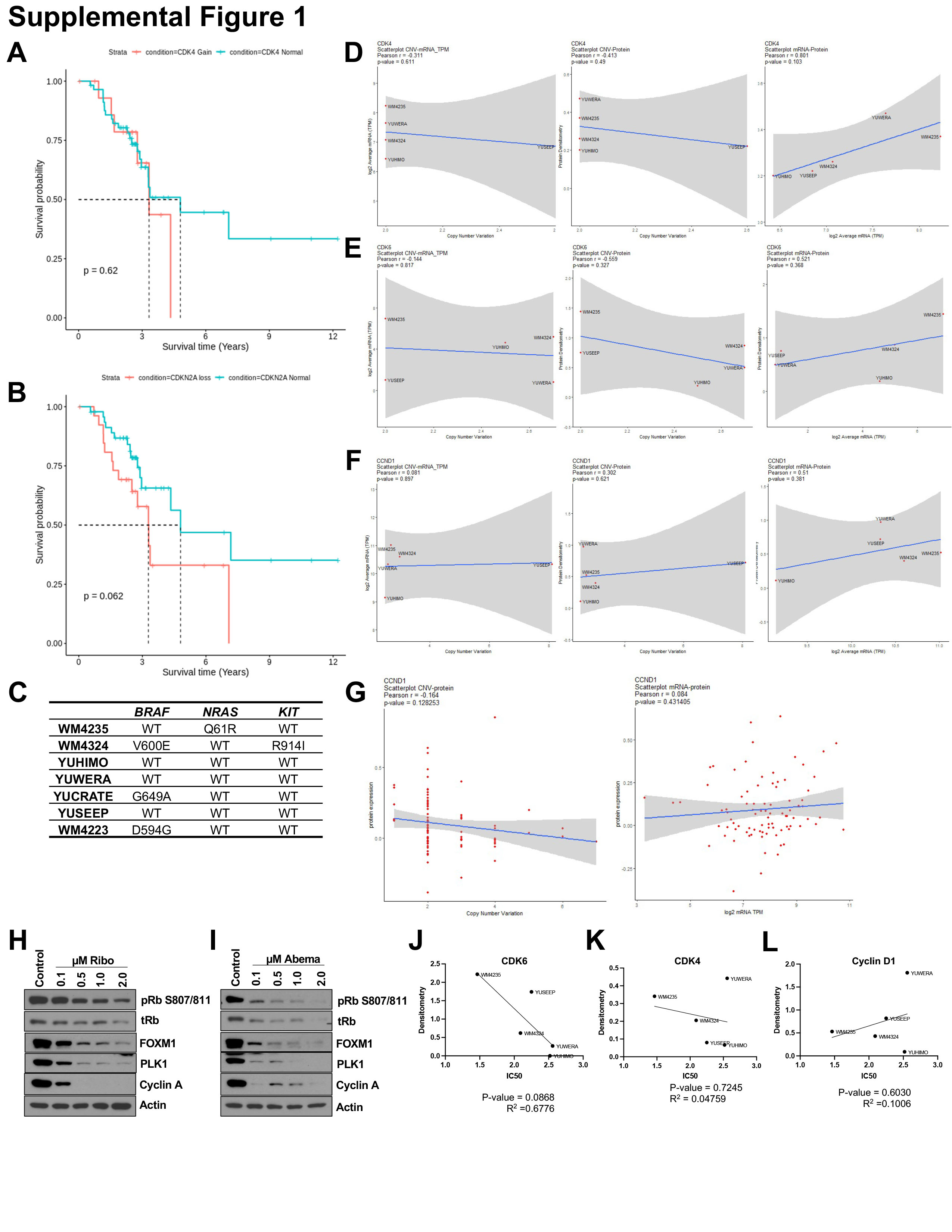

Supplement: Supplementary file 1 — Supplemental Figure 1 [file 41388_2023_2900_MOESM1_ESM.jpg]

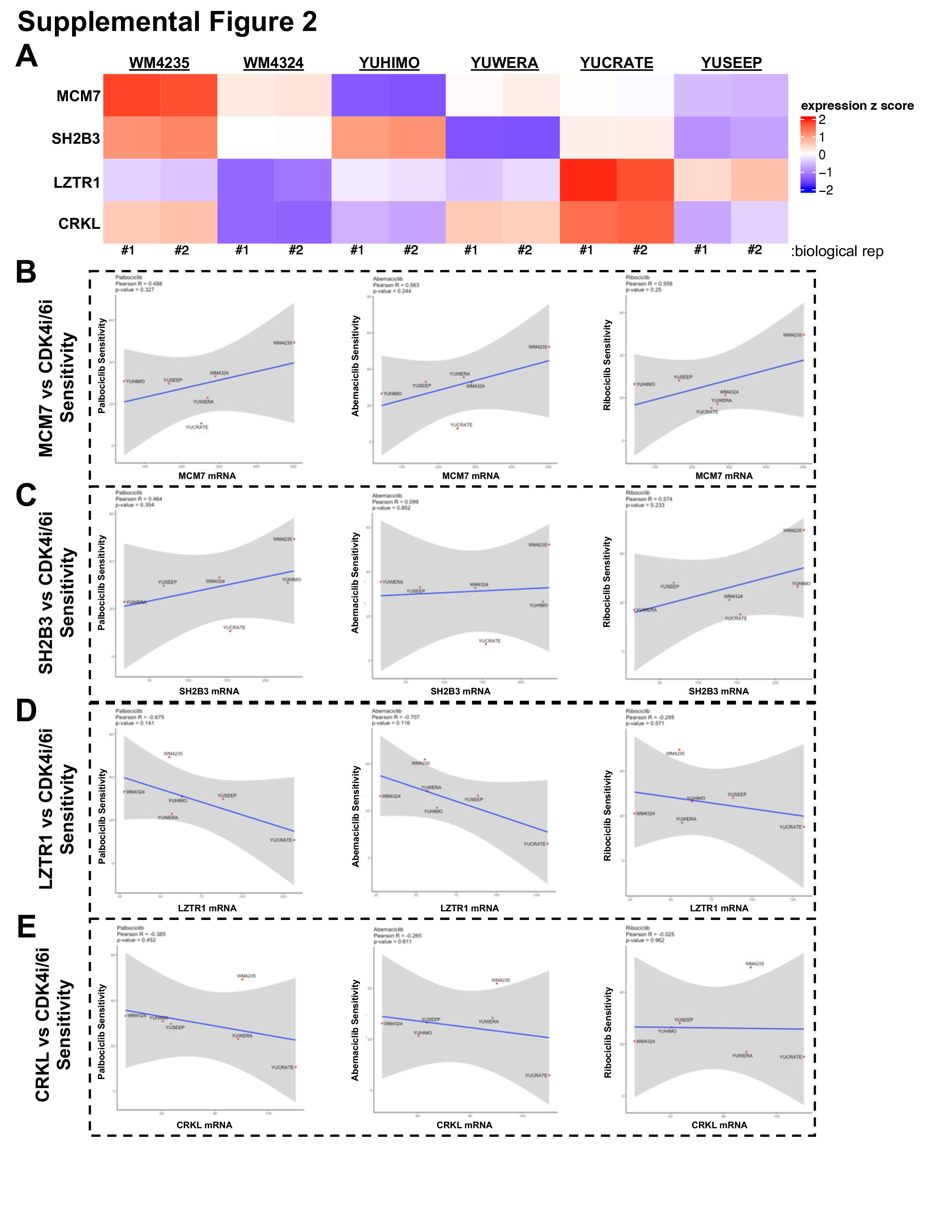

Supplement: Supplementary file 2 — Supplemental Figure 2 [file 41388_2023_2900_MOESM2_ESM.jpg]

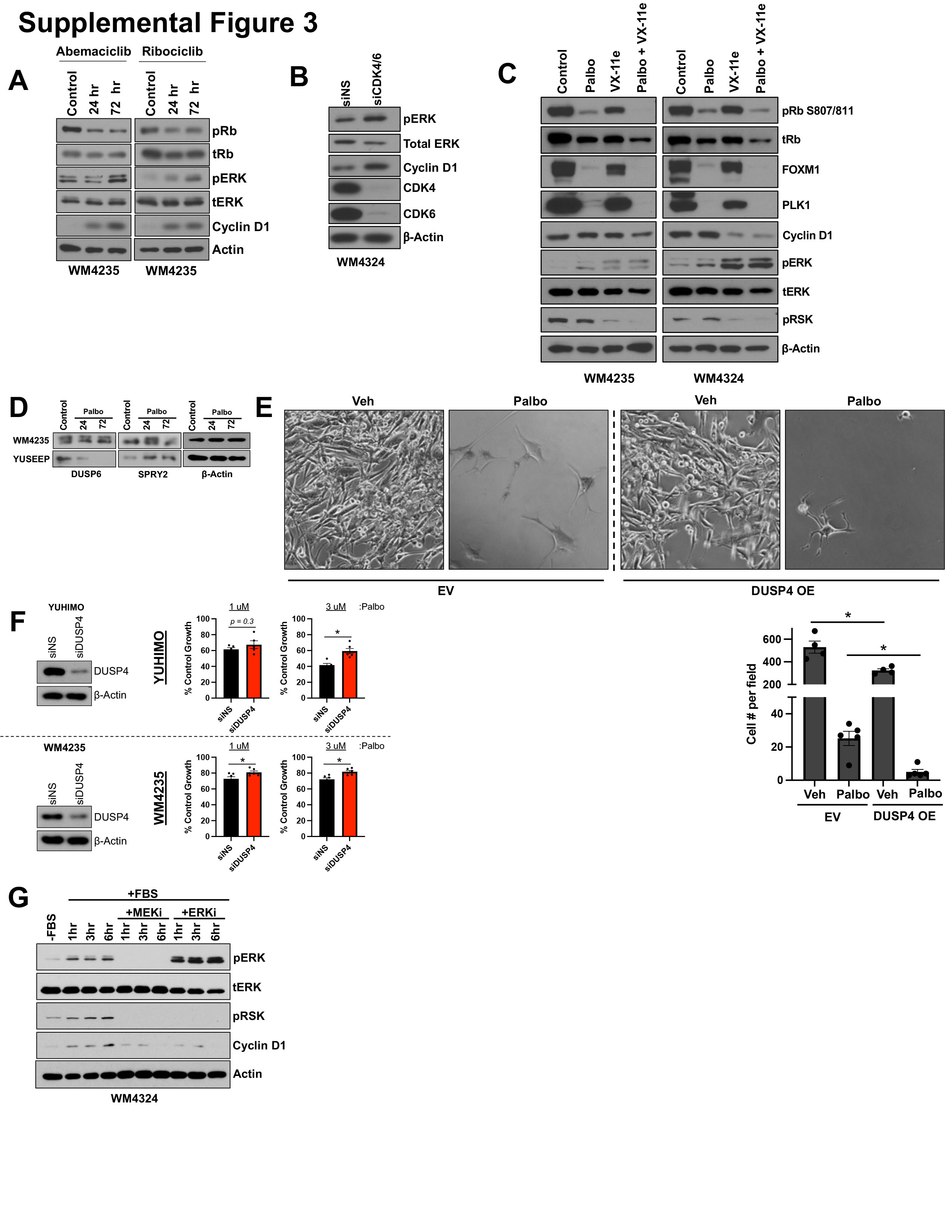

Supplement: Supplementary file 3 — Supplemental Figure 3 [file 41388_2023_2900_MOESM3_ESM.jpg]

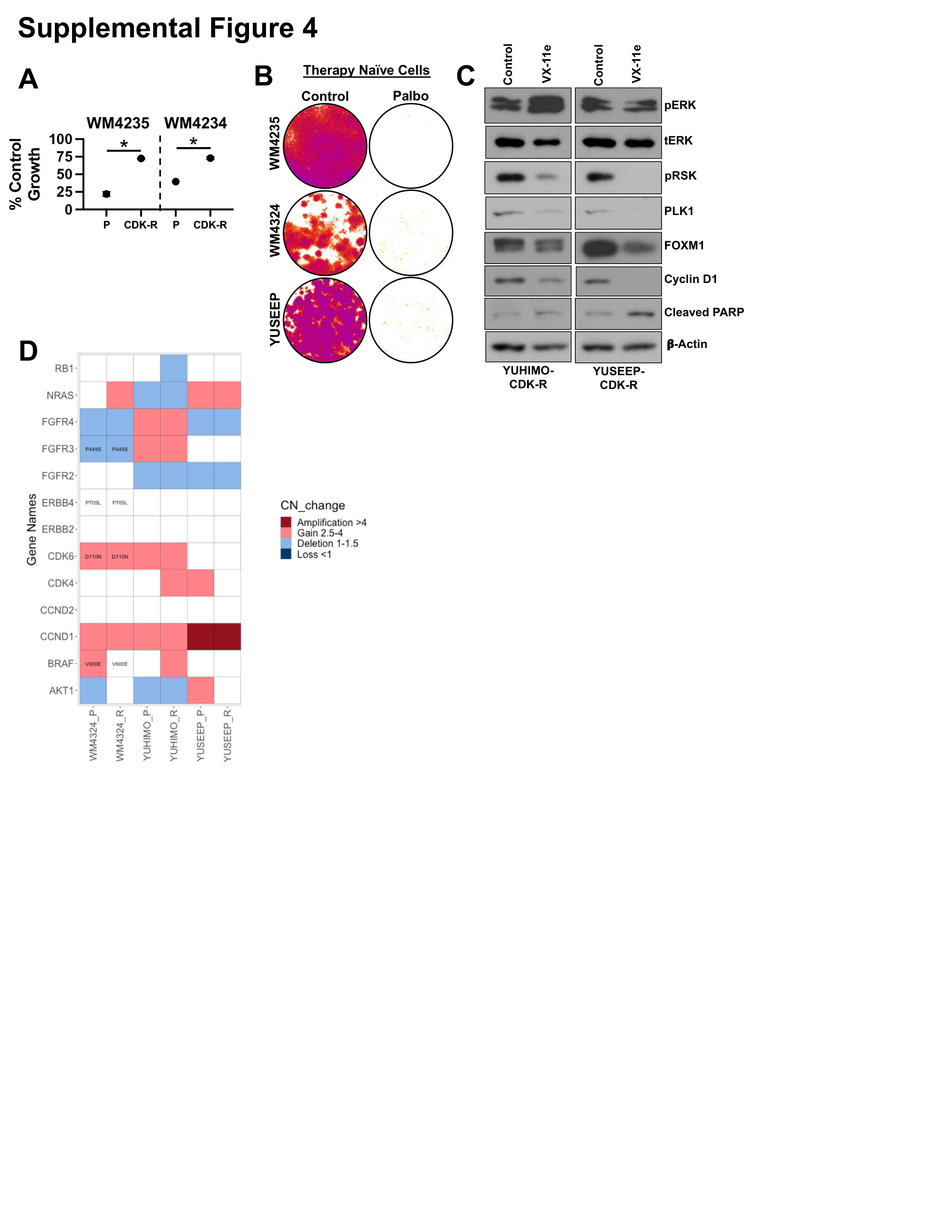

Supplement: Supplementary file 4 — Supplemental Figure 4 [file 41388_2023_2900_MOESM4_ESM.jpg]

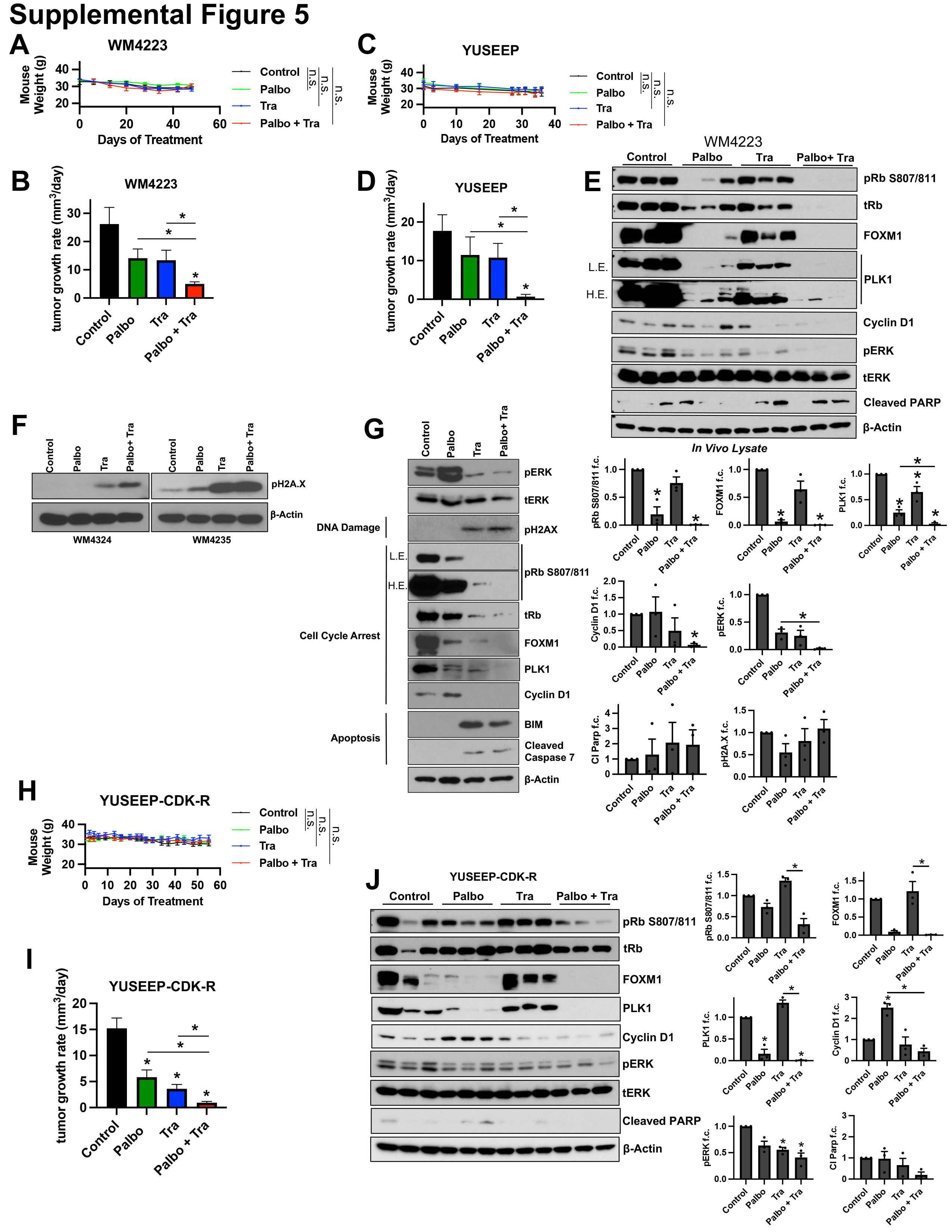

Supplement: Supplementary file 5 — Supplemental Figure 5 [file 41388_2023_2900_MOESM5_ESM.jpg]
