# Supplementary material for: ERK hyperactivation serves as a unified mechanism of escape in intrinsic and acquired CDK4/6 inhibitor resistance in acral lentiginous melanoma
Source: Oncogene. 2023 Dec 8;43(6):395–405. doi: 10.1038/s41388-023-02900-6 (PMC10837073; doi:10.1038/s41388-023-02900-6)
Supplement: Supplementary file 6 — Supplemental Figure Legends [file 41388_2023_2900_MOESM6_ESM.docx]

**Supplemental Figure 1:** (**A**) Kaplan-Meier estimator and log-rank test were used to compare survival probabilities between CDK4 CNV gain group (n=16) and CDK4 CNV wild type group (n=57), and (**B**) survival probabilities between CDKN2A CNV loss group (n=27) and CDKN2A CNV wild type group (n=47) of Acral melanoma patients. (**C**) Table of *NRAS*, *BRAF*, and *KIT* mutational status for the ALM cell lines used in this study. (**D**) mRNA TPM levels were plotted against copy number variation values, protein densitometric values were plotted against copy number variation values, and protein densitometric values were plotted against mRNA levels across 5 ALM cell lines for CDK4, (**E**) CDK6, and (**F**) CCND1. (**G**) RPPA protein values were plotted against both copy number variation and mRNA TPM values across skin cutaneous melanoma primary tumor samples in TCGA database (n=89). Pearson correlation tests were performed correspondingly. Large p values (>0.05) for regression coefficients of independent variables indicates there is not enough evidence to reject null hypothesis and there is no association between the changes in the independent variables (copy number variation, mRNA expression) and the shifts in the dependent variable (protein expression). (**H**) WM4324 cells were treated with ribociclib or (**I**) abemaciclib for 24 hours before immunoblotting for proteins shown. (**J**) Palbociclib IC50 values from Figure 1D were plotted against the densitometric values from Figure 1B of CDK6 (**K**) CDK4 and (**L**) cyclin D1 protein expression. Pearson correlation tests were performed correspondingly.

**Supplemental Figure 2:** (**A**) Baseline mRNA expression of the genes denoted acquired from 2 biological replicates for each cell line. (**B**) Tumor sensitivities to CDK4i/6i (Palbociclib, Abemaciclib, and Ribociclib respectively) were plotted against mRNA expression level of MCM7, (**C**) SH2B3, (**D**) LZTR1, and (**E**) CRKL across 6 baseline Acral melanoma cell lines and Pearson correlation tests were performed correspondingly.

**Supplemental Figure 3:** (**A**) Cells were treated with ribociclib (500 nM) before protein lysate was isolated and Western blotted. (**B**) Cells were transfected with non-specific siRNA (siNS) or siCDK4/6 for 72 hrs before protein lysate was isolated and Western blotted. (**C**) Cells were treated with palbociclib (500 nM) +/- VX-11e (300 nM) for 72 hours before lysate was Western blotted. (**D**) Cells were treated with palbociclib (500nM) and immunoblotted for DUSP6 and SPRY2. (**E**) Empty vector (EV) or DUSP4 was overexpressed (OE) in YUHIMO cells and YUHIMO cells were subsequently treated with palbociclib (500 nM) for 2-3 weeks. Brightfield microscopy shown, quantitation of 5 representative fields shown below images. * p<0.05, mean and s.e. shown. (**F**) Cells were treated with siNS or siDUSP4 for 24 hours prior to treatment with palbociclib (1-3 uM) for 72 hours before cell numbers were quantified using MTT (right). Western blotting confirmation of DUSP4 silencing (left). (**G**) Cells were serum starved for 24 hours before being stimulated with complete media in the presence or absence of trametinib (MEKi, 10 nM) or VX-11e (ERKi, 300 nM) for the timepoints shown before protein lysate was isolated and Western blotted.

**Supplemental Figure 4:** (**A**) Parental and CDK-R cells were treated with ribociclib (10 uM; 72 hrs) before cell number was quantified by MTT (**B**) Parental and respective CDK-R cells were treated with palbociclib (500 nM) for up to 4 weeks before colonies were fixed and stained with crystal violet. (**C**) Parental and CDK-R cells were treated with palbociclib (500 nM) +/- VX-11e (300 nM) for 72 hours before lysate was Western blotted. (**D**) Mutational and copy number data of the genes denoted in parental (P) and CDK4i/6i resistant (R) cell line pairs. *p<0.05, mean and s.e. shown throughout.

**Supplemental Figure 5:** (**A**) Weights of NSG mice implanted with WM4223 and treated with vehicle control, palbociclib plus or minus trametinib. (**B**) Calculated tumor growth rates across the treatment arms of mice implanted with WM4223 (n=5 for each treatment). (**C**) Weights of NSG mice implanted with YUSEEP and treated with vehicle control, palbociclib plus or minus trametinib. (**D**) Calculated tumor growth rates across the treatment arms of mice implanted with YUSEEP (n=5 for each treatment). (**E**) WM4223 tumor-bearing mice were treated for 72 hours with vehicle control, palbociclib, trametinib, or the combination of palbociclib + trametinib before mice were sacrificed and tumor lysate was isolated for Western blotting (n=3 for each condition). (**F**) Cells were treated with palbociclib (500 nM) and/or trametinib (10 nM) for 72 hrs before protein lysate was immunoblotted. (**G**) WM4324 cells were treated with palbociclib (500 nM) and/or trametinib (10 nM) for 10 days before protein lysate was immunoblotted. (**H**) Weights of NSG mice implanted with YUSEEP-CDK-R cells and treated with vehicle control, palbociclib plus or minus trametinib. (**I**) Calculated tumor growth rates across the treatment arms of mice implanted with YUSEEP-CDK-R (n=7 for the palbo + tra arm, n=6 for the other treatment arms). (**J**) YUSEEP-CDK-R tumor-bearing mice were treated with vehicle control, palbociclib, trametinib, or the combination of palbociclib + trametinib before mice were sacrificed at endpoint and tumor lysate was isolated for Western blotting (n=3 for each condition). *p<0.05, mean and s.e. shown throughout.
